# From protein-protein interactions to protein co-expression networks: a new perspective to evaluate large-scale proteomic data

**DOI:** 10.1186/s13637-017-0059-z

**Published:** 2017-03-20

**Authors:** Danila Vella, Italo Zoppis, Giancarlo Mauri, Pierluigi Mauri, Dario Di Silvestre

**Affiliations:** 1 Institute for Biomedical Technologies - National Research Council (ITB-CNR), 93 Fratelli Cervi, Segrate, Milan, Italy; 2 Department of Computer Science, Systems and Communication DiSCo, University of Milano-Bicocca, 336 Viale Sarca, Milan, Italy

**Keywords:** Co-expression network, -Omics data, PPI network, Systems biology, Topological analysis, WGCNA, Pearson’s correlation

## Abstract

The reductionist approach of dissecting biological systems into their constituents has been successful in the first stage of the molecular biology to elucidate the chemical basis of several biological processes. This knowledge helped biologists to understand the complexity of the biological systems evidencing that most biological functions do not arise from individual molecules; thus, realizing that the emergent properties of the biological systems cannot be explained or be predicted by investigating individual molecules without taking into consideration their relations. Thanks to the improvement of the current -omics technologies and the increasing understanding of the molecular relationships, even more studies are evaluating the biological systems through approaches based on graph theory. Genomic and proteomic data are often combined with protein-protein interaction (PPI) networks whose structure is routinely analyzed by algorithms and tools to characterize hubs/bottlenecks and topological, functional, and disease modules. On the other hand, co-expression networks represent a complementary procedure that give the opportunity to evaluate at system level including organisms that lack information on PPIs. Based on these premises, we introduce the reader to the PPI and to the co-expression networks, including aspects of reconstruction and analysis. In particular, the new idea to evaluate large-scale proteomic data by means of co-expression networks will be discussed presenting some examples of application. Their use to infer biological knowledge will be shown, and a special attention will be devoted to the topological and module analysis.

## Introduction

The development of systems biology approaches based on graph theory [1–3] is receiving a great boost by the improvement of the -omics technologies that allow more and more big amount of accurate qualitative and quantitative measures [4, 5]. New methodologies have also been developed to increase knowledge about protein-protein interactions (PPIs) [6]. As a result, the PPI networks combined with protein and with gene expression levels are today widespread to investigate biological systems [7–10].

The magnitude of -omics data provides the opportunity to decode in alternative way the role of biological molecules and processes characterizing the emergent phenotypes. In this scenario, a common procedure to evaluate gene expression levels is based on statistics that measure the dependence between variables, and the resulting co-expression networks are used to identify genes functionally related or controlled by the same transcriptional regulatory program [11–13]. Unlike gene expression levels, the use of proteomic data to infer co-expression networks has been explored through few studies [14–20]. Similar to PPI and gene co-expression networks, these networks have been evaluated at topological level in terms of edge rearrangement, as well as of modules associated with common cellular functions. Although different aspects including data collection and network reconstruction need to be improved, the preliminary results are proving this approach promising as alternative to evaluate large-scale proteomic data. This could have important effects into clinical applications favoring the way toward the use of multiple biomarkers and their relationships [17, 21–24]. Thus, in addition to improve basic research, these elements may contribute to develop most efficient diagnosis and prognosis methods to a more preventive, predictive, and personalized medicine [25–27].

Based on these premises, in this review we introduce the reader to PPI and co-expression networks. The recent idea to describe and to evaluate proteomic data by means of co-expression networks will be discussed presenting some example of application. Their use will be shown to infer biological knowledge, and a special attention will be devoted to the topological and module analysis.

## Protein interaction networks

**Fig. 1:**
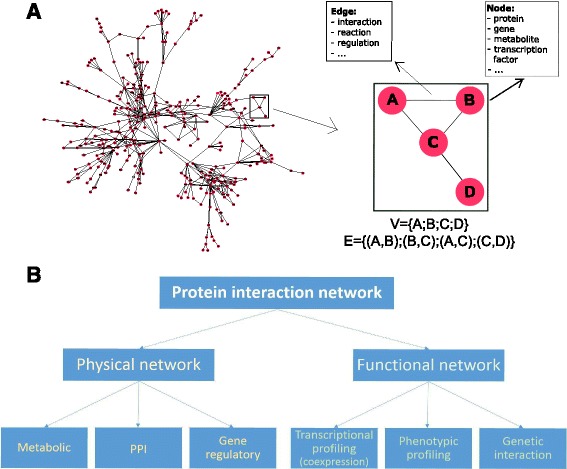
**a** Biological networks. Nodes may represent several types of biological elements, while the edges describe the nature of their relationship. If *A* and *B* are two nodes connected by an edge, (*A,B*)∈*E*, *B* is a *neighbor* of *A* or *A* and *B* are adjacent. **b** Protein network classification proposed by Vidal et al. [25]

Graph theory is a powerful abstracting machinery that allows to model several types of system, both natural and human-made, ranging from biology to sociology science [28]. A graph, also called network, provides a system representation in terms of relationships among the elements that make it up; a set of nodes *V*, stands for the elements of the system, while a set of edges *E*, stands for their relations. Mathematically, we refer to a graph as *G*=(*V,E*) (Fig. 1 a).

Concerning biological networks, the nodes may be correlated of attributes representing characteristics of interest, such as expression levels or GO terms. In the same way, the edges may possess attributes describing the relation between nodes, for example indicating the strength of the interaction or its reliability; edges may also be directed or undirected, and here we shall mainly deal with undirected edges. Using the framework described in Fig. 1, a protein interaction network is defined as a complex graph, where the nodes are proteins and the edges represent their relation, generally physical or functional, like proposed by Vidal et al. [25].

### PPI: physical and functional protein links

A protein interaction network usually refers to physical PPIs [29], but several meanings have been attributed to this term. In fact, a group of proteins working together to perform a biological function not necessarily are in direct contact, but their relation may be of regulation or influence, for example, making use of intermediary molecules. For this reason, the term PPI has not only been exclusively used to indicate a physical contact between proteins, but also proteins connected by functional links. It is important to bear in mind that proteins participate to physical-chemical connection depending on the biological context where they are [30]. Thus, the interactions composing a given network could not occur in any cell or at any time. However, if two interacting proteins are experimentally identified in a given sample, we assume they also interact in the system we are studying, thus their relation is reported in the reconstructed PPI network to be analyzed.

### PPI: detection, storage, and analysis tools

The main approaches to demonstrate physical interaction between proteins are the yeast two-hybrid (Y2H) method and the tandem affinity purification coupled with mass-spectrometry (TAP-MS) [6]. To reduce the identification of false interactions, these experimental data are complemented with computational methods of prediction [31–33]. Other methods are used to identify functional relationships, and most of them rely on protein expression data [20], analysis of gene co-expression patterns [34], and analysis of sequences or phylogenetic properties, as Rosetta Stone or Sequence co-evolution methods [35].

**Fig. 2:**
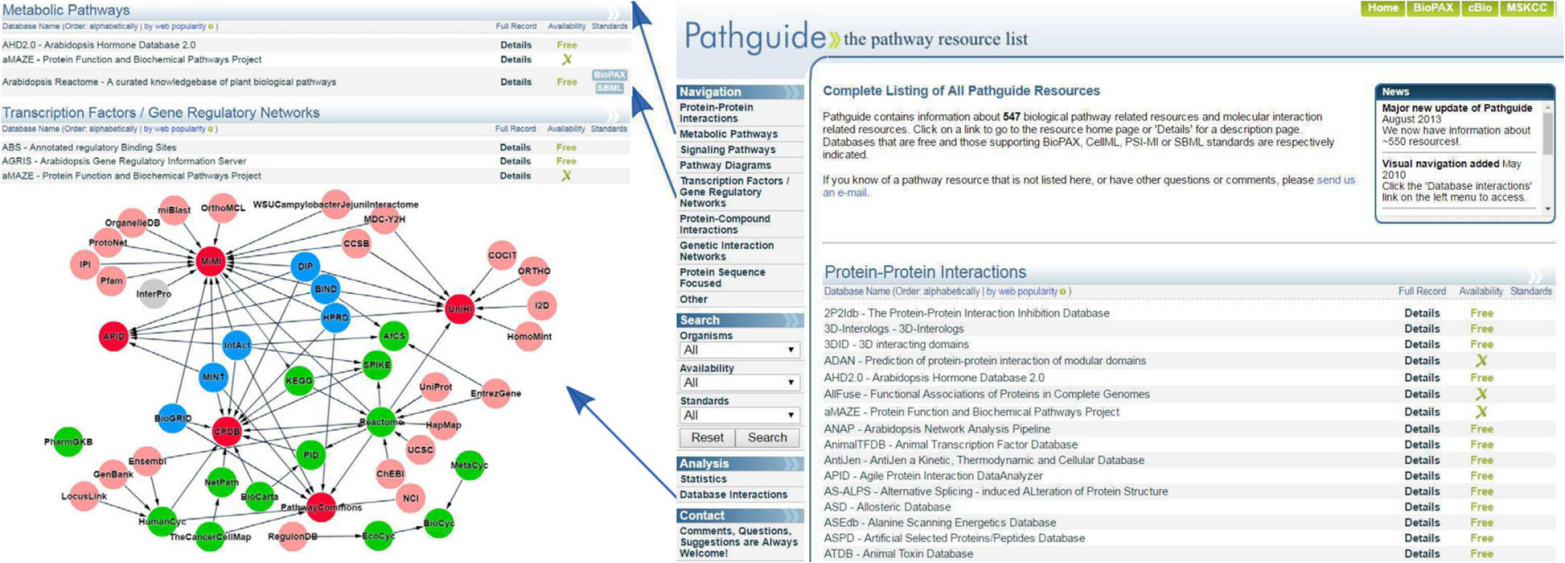
Pathguide website [40]. A repository containing information about 547 resources of molecular interactions and pathways

Both physical and functional PPIs are stored in public repositories. The most popular include MINT [36], IntAct [37], STRING [38], and HPRD [39]. The latter specifically collects interactions related to *Homo sapiens*, while other databases like STRING collect different kinds of interactions (from experiments/biochemistry, annotated pathways, gene neighborhood, gene fusion, gene co-occurrence, gene co-expression, and text-mining) and different organisms. A useful list of repositories presented by De Las Rivas et al. [29] provides a classification in categories (primary, meta, and prediction database) according to method used to detect interactions. Moreover, an exhaustive collection of more than 500 databases is available in the Pathguide website (Fig. 2) [40].

**Fig. 3:**
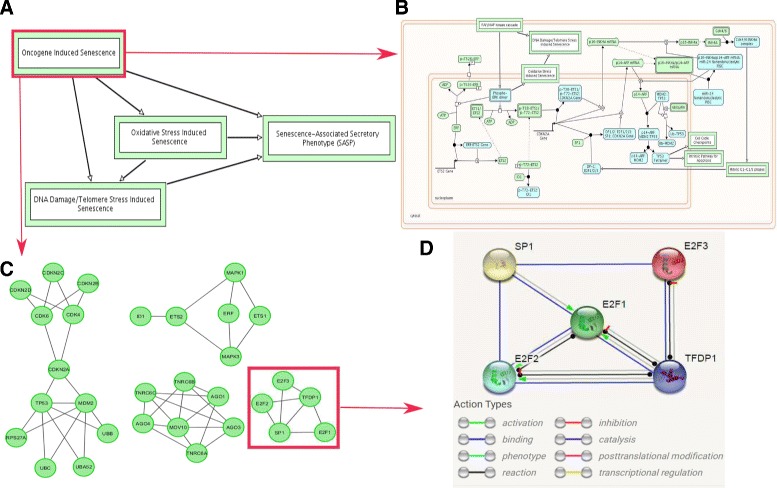
ReactomeFIViz: from disease pathway to PPI network. Main steps to obtain a protein functional and a physical protein network, starting from a specific pathway (oncogene induced senescence). Using ReactomeFIViz, pathways can be visualized in relation with others (**a**), can be detailed as a diagram showing all intermolecular relationships (**b**), and as a protein functional interaction network (**c**) showing just the relation among proteins that cooperate to perform a given molecular function. Finally, starting from a group of protein of interest, it is possible to obtain a network of protein-protein interactions by STRING; in the reported example, the interactions shown are limited to physical type, in particular binding, activation and inhibition (**d**)

The development of computational tools to retrieve, visualize, and analyze biological networks is a key aspect of the systems biology studies, like the production of accurate -omics data and the collection of reliable molecular interactions. The most broadly adopted softwares include Cytoscape and its plugins [41], VisANT [42], atBioNet [43], PINA [44], and Ingenuity [45] which represents a commercial solution. On the contrary, Cytoscape is a software now developed by an international consortium of open-source developers. Figure 3 shows a possible use of the ReactomeFIViz Cytoscape’s plugin to obtain networks (both functional and physical) associated with a given biological function. ReactomeFIViz is focused to pathways and patterns related to cancer and other pathologies [46]. This is of importance in the context of biomedical research, and detailed reviews about network models to investigate complex diseases have been published by Cho et al. [47] and by Vidal et al. [25]. Both works show how functional and physical links can be used to investigate disease mechanisms, and PPI networks emerge as effective model to evaluate different biomolecules acting in complex biological systems, thus providing an insight on phenomenons involved in a given physio-pathological context.

## Co-expression networks

The great amount of data produced by microarray and RNA-seq technologies has driven the need of methods to objectively extract meaningful informations, such as genes differentially expressed or sharing a similar expression pattern. A widely adopted approach to evaluate transcript levels is based on statistics that measure the dependence between variables [48]. Co-expression represents the first step of inference that defines a relation between pairs of transcripts. It is based on the concept that transcript profiles of time series, or result of specific perturbations, may be indicative of dynamics and differences between transcripts, implying their regulation. Following the processing of transcript levels, the result is a co-expression network defined as an undirected graphs where the nodes correspond to genes, and the edges indicate significant co-expression relationships, but not causality. This aspect is faced in the context of transcriptional regulatory networks [49], where pairs of genes are considered in a systemic perspective of cooperation, including co-regulation, activation/suppression, and indirect control through the action of siRNA, miRNA, proteins, metabolites, and epigenetic mechanisms. This complexity make difficult the inference of transcriptional regulatory networks by using exclusively transcriptional profiles. In fact, in addition to co-expression, next levels of inference require more information and different modeling techniques, including Boolean networks, Bayesian networks, or differential equations (ODEs), which are revised in more detail in studies addressing *reverse engineering* approaches [49].

**Fig. 4:**
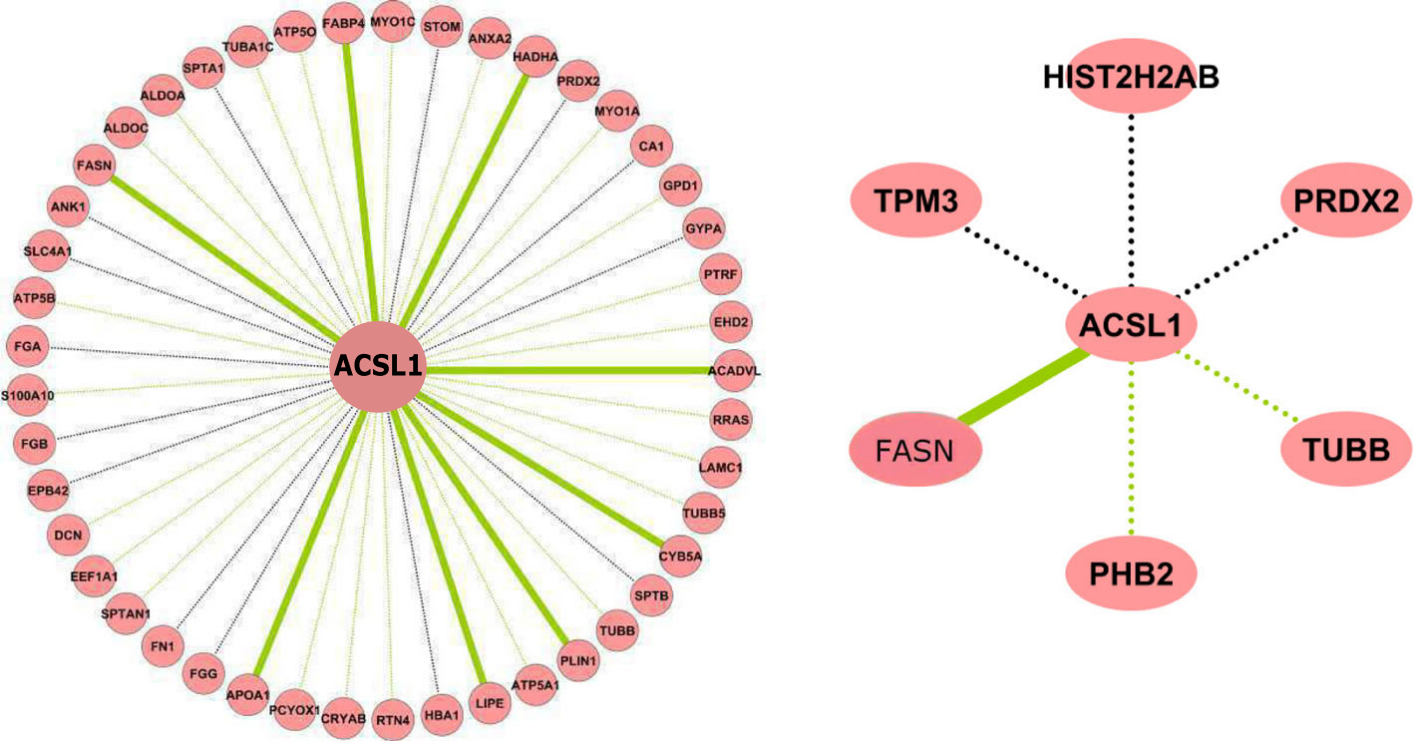
The figure shows the ACSL1 protein and its neighbors in two co-expression networks obtained by processing the protein expression profiles of a control group and a group of patients affected by amyloidosis disease. In the considered groups of samples, ACSL1 shows a different degree. It suggests that this protein may have a key role in the emergent phanotypes. *Green edges*represent a positive correlation between the expression profiles, while *black edges*indicate negative correlations. The *thick edges* indicate known interactions present in public repositories as PPI

Gene co-expression networks are topologically analyzed to identify hubs/bottlenecks and node communities sharing high co-expression score; communities are the starting point to identify topological, functional, and/or disease modules related to specific biological phenotypes [50, 51]. Different studies have shown that genes functionally related, and sharing Gene Ontology (GO) terms, usually present higher co-expression score [52]. Moreover, variations of the co-expression score are evaluated to select topological relevant nodes whose number of interactions changes under specific conditions or perturbations [18] (Fig. 4).

In the last 10 years, the improvement of the liquid chromatography and the mass spectrometry has given a great boost to large-scale proteomics analysis, making available the expression profiles of thousands of proteins per sample [53]. Due to the similarity between gene and protein matrices, the use of proteomic data to infer protein co-expression networks has been recently explored to investigate the role of proteins in specific physio-pathological contexts. Although different aspects need to be improved, this approach takes into account protein relationships, and, with respect to conventional methods, it represents an alternative to gain a deeper insight of the protein characterizing a given system. This issue will be discussed with greater detail in the paragraph 5.

### Aspects of construction

**Fig. 5:**
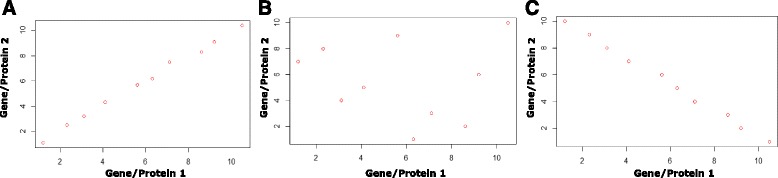
Possible cases of correlation between two variables. **a** Positive correlation. **b** No correlation. **c** Negative correlation

To build a co-expression network, an important aspect concerns the computation of a co-expression score, which weigh the correlation of two genes/proteins in response to the considered conditions (Fig. 5). To address this issue, metrics to measure gene/protein co-expression have to be considered (Table 1); the most used metrics include Pearson’s correlation (PC), Spearman’s correlation, Kendall’s correlation, and mutual information [48, 54]. Various methods have been also proposed to define proper thresholds to select significant relations. Some of them are based on statistical analysis [55] and on network properties [56], while other interesting approaches aim to minimize the false positive links [57]. Finally, not less important is the selection of appropriate experimental samples/conditions to be processed. A condition-independent analysis is used to find relations of co-expression actual in different biological contexts; on the contrary, a condition-dependent analysis aims to find relations associated with specific phenotypes.

**Table 1:** Measures of dependence between two variables

Co-expression measures	What measures?	Input/Output	Features
Pearson’s correlation (PC)	Tendency to respond in opposite/same direction across different samples	Input: gene expressions value Output: • [0,1] both genes increase • [−1,0] one increase and other decrease	• Sensitivity to outliers • Bad array of expression level can determine positive PC value • Measure linear relations
Spearman’s correlation (SC)	Tendency to respond in opposite/same direction across different samples	Input: ranking values from expression levels in samples Output: • [0,1] Both genes increase • [−1,0] One increase and the other decrease	• Robust to outliers • Detect non-linear associations
Mutual information	Reduction of uncertainty of a gene given the knowledge about other gene	Input: gene expression values Output: • 0 there is no interdependence • >0 there is interdependence	• Measure complex non-linear type relations (rarely present in biological data) • More samples are needed than PC, SC • Time-consuming computation
Kendall	Correspondence/compatibility among two rankings	Input: gene expression value Output: • 1 perfect correspondence • -1 rankings exactly inverted	• Similar to SC • Robust to outliers • Assumes fewer values than SC in the range [−1,1]

The co-expression score computation may be faced by using any statistical or computational tool that allows to evaluate the dependence between variables. Some tools have been specifically designed to construct, visualize, and analyze co-expression networks. For example, the ExpressionCorrelation Cytoscape’s plugin allows to process microarray data and provides a similarity matrix computed by PC [58]. In addition to being user-friendly, the main advantage of this tool is that the reconstructed networks are directly imported in Cytoscape where it may be evaluated by other plugins.

WGCNA is one of the most used approaches to build and to analyze gene co-expression networks [59], and it has been recently adapted for proteomics use also [14–20]. It provides a weighted network model by converting a co-expression measure to a connection weight. The network is fully specified by an adjacency matrix, where the component *a*_ij_ defines the strength of connection between nodes i and j. The value of *a*_ij_ is computed through the co-expression similarity *s*_ij_ (1), defined as the absolute value of correlation among the profiles of nodes i and j. It can be defined in two ways: to obtain an unweighted network, the *s*_ij_ is filtered by a threshold *τ* such that *a*_ij_ takes on value [0,1] (hard-thresholding) (2), while to obtain a weighted network *a*_ij_ is defined by a power adjacency function (soft-thresholding) (3):

1 $$ {s}_{\text{ij}} = \vert{\text{cor}(i,j)}\vert  $$

2 $$  a_{\text{ij}} = \left\{ \begin{array}{rl} 1 & s_{\text{ij}} \ge \tau \\ 0 & s_{\text{ij}} < \tau \\ \end{array}\right.  $$

3 $$ {{a}_{\text{ij}}} = s_{\text{ij}}^{\beta}  $$

The R WGCNA package provides the possibility to use different types of metrics, including Spearman’, Pearson’, Kendall’s correlation (see function *cor*), and the biweight midcorrelation (see function *bicor*) [60]. Spearman’s correlation is a non-parametric measure of correlation. Pearson’s correlation can be used when data are normally distributed, but it is quite susceptible to the presence of outliers. In this case, the biweight midcorrelation is recommended because it is more robust to outliers. The package allows to compute both the correlation and the Student *p* value for multiple correlations in case of missing data (see function *corAndPvalue* and *bicorAndPvalue*), while the function *qvalue* computes the *q* value to measure the significance of each feature in terms of false discovery rate rather than false positive rate [61]. The unweighted network displays sensitivity to the choice of the correlation values cut-off, thus, it is important to use a proper criterion to select the edges to include in the network. It is important to take into account the correlations are computed among each pairs of genes/proteins leading to a high rate of false positive values. Thus, to build an unweighted network and to reduce the inclusion of not significant correlations, it is recommended to set a cut-off also for *p* and *q* values. Concerning the weighted networks, the choice of the *β* parameter is based on the scale-free topology criterion [62]. This method represents an improvement over unweighted networks based on dichotomizing the correlation matrix; the continuous nature of the gene co-expression information is preserved, and the results of weighted network analyses are highly robust with respect to the choice of the parameter *β* (soft-thresholding power). However, this thresholding method is based on the assumption that the network follows a scale-free topology, a hypothesis weak in some cases, as discussed in Section 4.1.

### WGCNA and proteomic issues

When the WGCNA is applied to proteomic or to metabolomic data, the choice of the optimal cutting parameters should be evaluated in relation to the nature of the data analyzed. In fact, due to the low coverage of the current analytical technologies, the produced dataset are often incomplete, and the methods need to be properly modified [63]. A major concern is the high rate of missing values that introduce loss of information and significant bias. To address this issue, several approaches including *K* nearest neighbor, least square methods, or local least square methods have been proposed for proteomic and metabolomic datasets too [64]. In other cases, a very simple approach has been adopted, such as the removal of all species with a number of missing data bigger than a given threshold [65]. However, to implement a more accurate analysis, it is recommended to process data by using an imputation method taking into account the nature of missing data. Three types of missing value have been identified: MCAR (missing completely at random), i.e., due to stochastic fluctuations in a proteomic dataset, MAR (missing at a random), i.e., due to multiple minor errors, and MNAR (missing not at a random), i.e., due to limits of abundance of peptides/proteins that instruments are able to detect. In general, methods work fine when a low percentage of missing value (≤10%) is present, but this threshold could be different in relation to the missingness mechanisms and imputation approach used [63, 64].

In addition to missing value, another important step of proteomic data preprocessing concerns their normalization [66]. Batch effects may occur in datasets run in different days or by different technicians. This phenomenon may increase by using isotope reagents which allow the quantitation of a limited number of samples, thus, preventing a simultaneous analysis of multiple samples which could reduce data heterogeneity. For these reasons, an appropriate data transformation is a prerequisite to capture true correlations. Also in the case of protein co-expression, valid correlations have to be selected by applying proper thresholds. To date, the most applications of WGCNA method on proteomic datasets used a the soft-thresholding, which defines the *β* value according the scale-free criterion [15, 16, 65]. However, since the application of WGCNA to proteomic dataset is a recent issue, and literature reports, few examples, the future evaluation of hard-thresholding approach might be useful.

## Network topological analysis

**Fig. 6:**
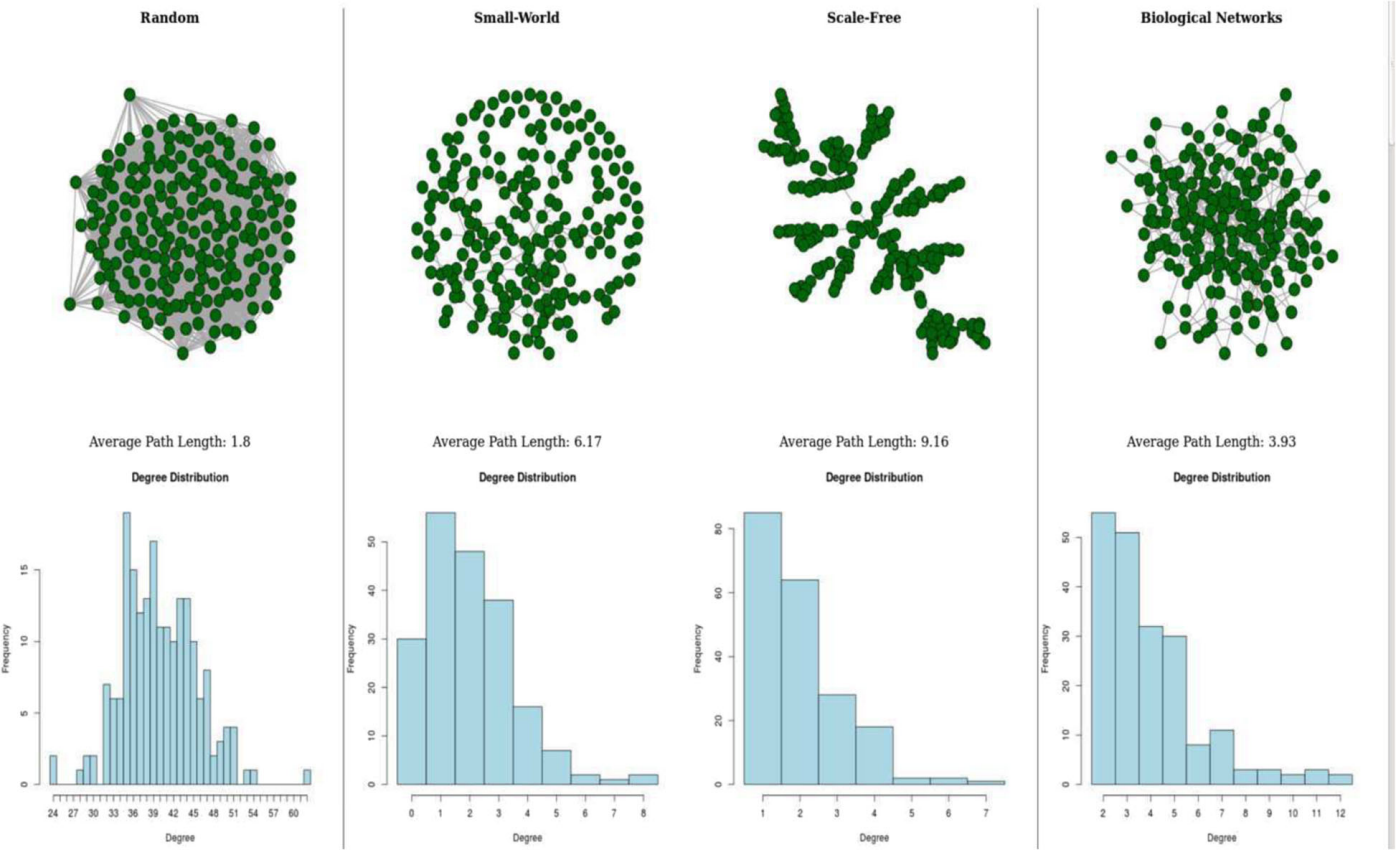
Shape and degree distribution of random, small-world, and scale-free model with respect to a biological network. Models were calculated by ELIXIR web tool [131]

**Fig. 7:**
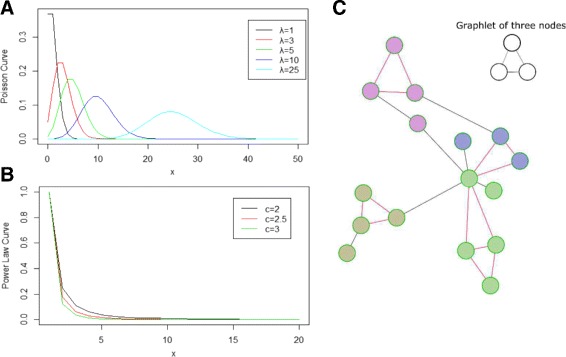
Functions used to describe the degree distribution of biological networks. Poisson curve **a** and power-law **b** shown for different parameters. **c** Example of graphlet of three nodes with frequency equal to 5

The structure of biological networks is closely related to the biological functions performed by a system (cell or tissue) under a given condition. Starting from this point, many studies aim to face biological questions by investigating the network models in terms of topology [67] and modular properties [68]. As for theoretical mathematical models proposed to describe the biological networks, the most claimed are Erdös–Rényi random graphs [69] and scale-free [70] (see Fig. 6). Other models, such as the geometric random graph (GEO) [71] and the small-world [72], have recently been proposed. In the context of biology, the random graph, proposed in 1950, has been overtaken by the scale-free model; in fact, the degree distribution of the scale-free model is a power-law curve that fits better than Poisson curve (typical of random graphs) the degree distribution of the experimental networks [70] (Fig. 7). Based on power law distribution, most nodes have a degree value far from the mean; specifically, most nodes have a low number of interactions while few nodes have a high number of interactions. These features lead a network structure less vulnerable and make the related system biologically robust [73]. Of note, the degree distribution may reflect the different role of proteins/genes, and those with a highest number of connections, so-called hubs, have a higher probability to be more biologically relevant than others. In other words, removal or modification of hubs may induce stronger alteration of the system equilibrium rather than removal or modification of nodes with low degree [74].

Although some topological properties are well described by a theoretical model, it may not be enough to affirm that the model represents well the real-world network considered [75]. For example, a study on PPI network of *Drosophila Melanogaster*and *Saccharomyces Cerevisiae*showed that the degree distribution was in agreement with scale-free model, but diameter, cluster coefficient, and graphlet frequency were closer to GEO [76]. Of note, based on graphlet frequency, the comparison among scale-free, random graph, and GEO models has shown a higher agreement of GEO with PPI network from eukaryotic organism [77, 78]. A possible reason of these findings is that the scale-free model fits networks that emerged from a stochastic growth, not subjected to an optimization process; while, PPI networks emerge from stochastic processes, and their structure is influenced by the evolutionary optimization that living systems have gone through [76].

Another model used to describe the PPI networks is the small-world. Like the random graph model, it is characterized by a Poisson curve. In a study focused on the investigation of proteins regulating the fat storage, the corresponding PPI network had a degree-distribution close to a Poisson curve rather than a power-law [79]. Moreover, the network showed a low average path length and a high clustering coefficient typical of small-world model. These parameters indicate a network organized into communities, like observed in PPI networks [80]. The small-world model preserves a modularity structure, and it is not characterized by hub nodes making the small-world networks more robust in the case of removal or modification of any node [73].

The topological evaluation of gene co-expression networks has shown that they are characterized by small-world and by scale-free properties, similar to many other real-world networks [81]. A study showed that the co-expression networks generated from large datasets are scale-free, but with an average clustering coefficient of several orders of magnitude higher than expected for similarly sized scale-free networks [82]. These opposite findings could be explained by the evidence that the topological properties of the co-expression networks may be influenced by different parameters, including the expression data or the similarity measures to evaluate the dependency between variables.

### Topological analysis

A key point of topological studies is the definition of mathematical models and metrics to describe the network’s properties and to select the most relevant nodes and substructures that may be of biological significance. Generally called centralities, metrics can be subdivided into measures related to nodes, edges, or whole network. Table 2 lists the main basic centralities used in the network topological analysis [83].

**Table 2:** Centralities calculated by the CentiScaPe Cytoscape’s plugin

Centrality	Description	Biological meaning
Diameter^a^	Defines the longest shortest path in the network	
Average distance^a^	Defines the mean length of all the shortest paths in the network	
Degree^b^	Describes the number of neighbors a node has	Highlights the number of nodes that regulated/regulate the node *v*
Eccentricity^b^	Describes the longest shortest paths a node develop, giving us a proximity information	Highlights the easiness of a protein to reach/to be reached by all the other proteins in the network
Closeness^b^	Describes, for the node *v*, the minimal sum of all the distances in the network	Highlights the probability of a protein to be functionally relevant for several proteins, but irrelevant for a few others
Radiality^b^	Describes the integration of a node into the network	Highlights the ability of a protein to be functionally relevant for several proteins, but irrelevant for a few others
Centroid^b^	Describes the neighborhood of nodes by highlighting nodes that have the highest number of neighbors separated by the minimal shortest path	Highlights a protein that tends to be functionally capable of organizing discrete protein clusters or modules
Stress^b^	Describes the number of shortest paths that pass through a node	Highlights the relevance of a protein as functionally capable of holding together communicating nodes
Betweenness^b^	Describes, for each couple of nodes, the number of shortest paths that pass through a specific node	Highlights the relevance of a protein as functionally capable of holding together communicating nodes
Bridging^b^	Describes the neighborhood of nodes by highlighting nodes with a high number of high-degree neighbors	Highlights a protein possibly bringing in communication sets of regulatory protein
Eigenvector^b^	Describes a sort of weighted degree, where not only the number of the neighbors is important but also the Eigenvector of the neighbors itself	Highlights a protein interacting with several important proteins, suggesting a central super-regulatory role or a critical target of a regulatory pathways
Edge betweenness^c^	Describes, for each couple of nodes, the shortest paths that pass through a specific edge	Highlights the relevance of the interaction as capable of organizing regulatory process

For each centrality, it is described the topological and biological meaning. The ^a^ indicates network’s properties. The ^b^ indicates node’s properties. The ^c^ indicates edge’s property

In the context of network organization, these centralities facilitate the answer to question about which proteins are most important and why. To give an idea of such analysis, we say that a vertex (i.e., a protein) is important (or central) if it is close to many other vertexes. There are many number of different centrality measures that have been proposed in literature but probably the most applied, and simple, is called *vertex degree*. The degree *d*(*v*) of a vertex *v*, in a network *G*=(*V,E*), counts the number of edges in *E* incident upon *v*. Given *G*, define *f*(*d*) to be the fraction of vertexes *v*∈*V* with degree *d*(*v*)=*d*. For different *d*_1_,*d*_2_,…,*d*_*n*_, the collection {*f*(*d*_1_),*f*(*d*_2_),…,*f*(*d*_*n*_)} is called the degree distribution of *G*.

A useful generalization of degree is the notion of vertex strength, which is obtained simply by summing up the weights of edges incident to a given vertex. The distribution of strength is sometimes called the weighted degree distributions defined in analogy to the ordinary degree distribution.

Another centrality measure widely used is known as betweenness [84]. It can be defined as follows: this measure summarizes the extent to which a vertex is located “between” other pairs of vertexes. In this case, centrality is based upon the perspective that importance relates to where a vertex is located with respect to the paths in the network graph. In other terms, betweenness centrality is based on communication flow. Nodes with a high betweenness centrality are interesting because they lie on communication paths and control information flow. Also called hubs/bottlenecks [85], they can represent important proteins in signaling pathways and can form targets for drug discovery. For example, by combining this data with interference analysis, targeted attacks on protein-protein interaction networks have been simulated to predict which proteins were better drug candidates [86].

Formally, betweenness can be defined as

4 $$  \text{Cl}(v) = \frac{\sigma(s,t | v)}{\sum_{s \neq t \neq v \in V}{\sigma(s,t)}}  $$

where *σ*(*s,t*|*v*) is the total number of shortest paths between *s* and *t* that pass through *v*, and *σ*(*s,t*) is the total number of shortest paths between *s* and *t* (regardless of whether or not they pass through *v*).

Other centralities used to globally evaluate the structure of a network include:

- Degree distribution: a function describing the proportion of nodes related to each observed degree
- Modularity: evaluates the presence of modules, such as a group of nodes characterized by the tendency to form more connections within the group than outside [87]
- Cluster coefficient: the ratio of the number of edges among a node and its neighbors and the maximum possible number of edges among all of them [72]
- Motif/graphlet frequency: evaluates the presence of small subgraphs with a specific pattern that appear in a real-world network more frequently than in the relative random network [88]
- Edge clustering coefficient: the ratio between the number of triangles (three nodes connected by three edges) including an edge, and the maximum number of possible triangles may include the edge [89]
- Maximal Clique centrality: a property of a node taking into account the cliques (i.e, a subgraph in which each pair of nodes is connected) including the node [90]

The simplest way to perform a network topological analysis by evaluating these centralities is through Cytoscape’s plugins, such as CentiScaPe [83] and NetworkAnalyzer [91], that provide the main basic methods to compute the topological properties of nodes, edges, and networks, both directed and undirected. Moreover, new plugins implementing recent developed topological centralities are CytoNCA [92] and CytoHubba [90].

### Module analysis

**Fig. 8:**
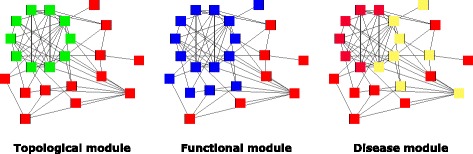
Example of topological, functional and disease modules not fully overlapped. The *green* nodes indicate a topological module, the *blue* nodes indicate a functional module, while the *yellow* nodes indicate a disease module

Regardless of the approaches used to obtain a network, the detection of protein/gene modules is of great interest because they represent the functional units at the base of the mechanisms responsible of the cellular life. In biological networks, the term module has acquired three meanings: topological, functional, and pathological/disease. The analysis of the network structure allows to detect the topological modules defined as group of nodes highly interconnected [68]. These nodes are often related to well-defined molecular functions, thus, their detection PPI networks can help to identify functional modules [93], defined as a group of functionally related proteins/genes highly connected by genetic/physical interactions, co-expression, as well as membership of the same molecular complex or biological pathway [94]. The comparison between pathological and physiological conditions has finally led to the definition of disease modules, such as a set of nodes with a putative key role concerning mechanisms impaired due to disease [26, 51]. Topological, functional, and disease modules are generally not fully overlapped and often a single topological module can be linked to different functional or disease modules or vice-versa (Fig. 8).

Due to the complex connectivity of the biological networks, the identification of modules is a challenging task. Various methods have been proposed, and most of them are exclusively based on network topology. Some representative examples include the betweenness-based method [95], the modularity optimization method [96], the spectral partitioning method [97], the core-attachment based method [98], and the graph-theoretic approach relying on cliques [99] and other topological properties [100]. To improve the accuracy of module detection, the integration of functional information is more and more used [101–104]. These methods exploit the GO terms which in some cases are used to compute a similarity score that measures the edge weight and drives the module detection [105, 106].

**Fig. 9:**
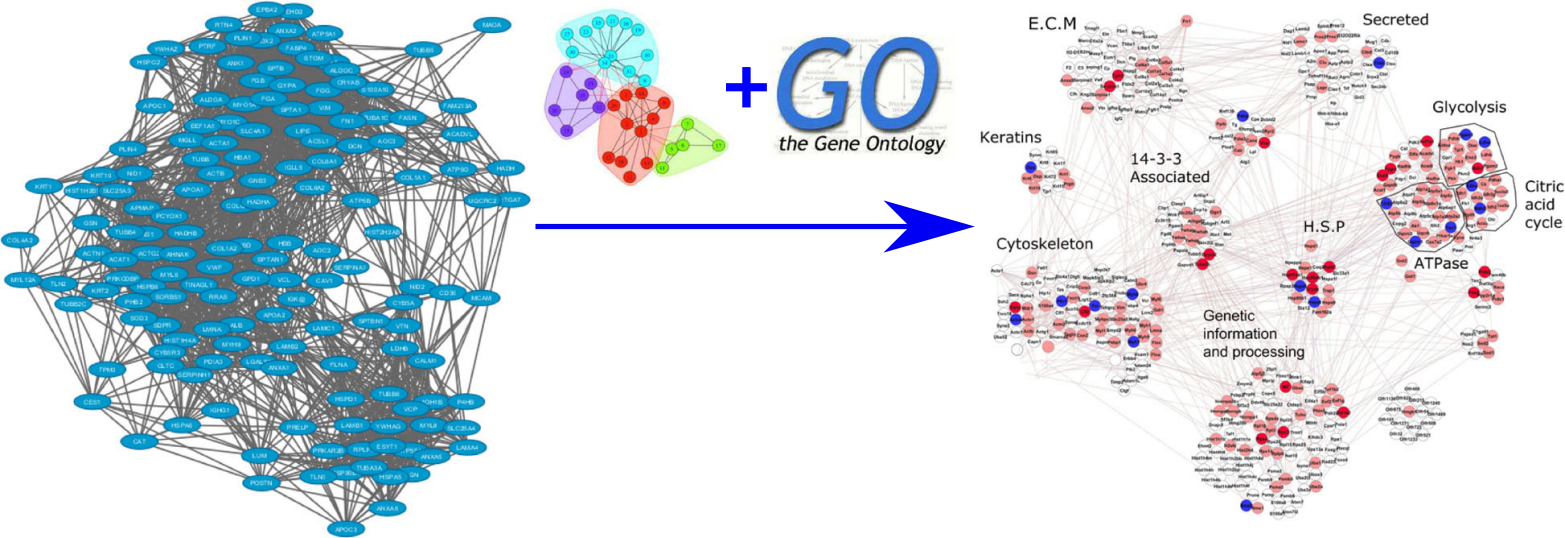
Procedure used to identify/predict modules in biological networks. The network structure is used to identify groups of highly connected nodes by graph clustering algorithm, while the GO annotations are used to improve the accuracy of the cluster prediction. The final result are clusters of nodes highly connected and related to functions/processes significantly enriched, thus acting at the basis of the emergent phenotypes

The GO term enrichment analysis is routinely used also after the module detection to assess their biological relevance [107, 108]. Making use of statistical tests, these approaches evaluate if genes/proteins of a module are enriched in common functional properties (Fig. 9). During this process, standard methods treat each gene/protein as an isolated objects. However, in the last few years some network-based enrichment approaches have emerged taking into consideration also the interactions among molecules [109–111].

The commonly used methods for module detection have been extended to co-expression networks to evaluate the conditional patterns of co-expression and to provide insight into the cellular processes underlying the emergent phenotypes. Since genes could be co-regulated only across a subset of phenotypes, a biologically-motivated clustering method should be able to detect these patterns. This issue is faced by biclustering algorithms which clusterize both genes and experimental conditions. They are widely studied, and many different approaches have been published and applied to identify genes regulated in a state-specific manner [112].

In the context of module detection, the WGCNA package also provides a procedure consisting of a hierarchical clustering algorithm based on a distance matrix calculated by similarity measure between gene/protein pairs [59]. After assigning nodes to modules, an aggregate module signature, called eigenvector, is computed; it can be considered as an object representing the expression profiles of the molecules belonging to the module, thus, it simplifies the comparison of different modules [113]. A wide range of tools to perform module analysis are available. They include several Cytoscape’s plugins, such as ClusterOne [114] and MCODE [100] and the Markov Cluster Algorithm (MCL) [115] or CFinder [99]. For a detailed view of these tools, the review by J.Ji et al. [116] is recommended.

## Studies related to the use of protein co-expression networks

The investigation of proteomic data by co-expression-based approaches has been first addressed by Gibbs et al. to infer the protein abundance and to overcome issues linked to peptide-protein mapping [14]. Starting from experimental datasets obtained by LC-MS, and by using a method derived from WGCNA, the authors proposed a protein co-expression network approach (ProCoNa) where the nodes are peptides and the edges are calculated by processing their intensity. The modules computed by co-expression analysis were strictly correlated with the investigated phenotypes and showed a significant enrichment of some GO terms. Following these findings, the authors explored the relationship between co-expression networks reconstructed from transcriptomic and proteomic data [15]. In this study, concerning SARS-CoV infection, they used a bipartite graph analysis to evaluate phenotype associations, overlaps, and module correlation, thus, providing a foundation of a true multi-omics signatures.

The idea to use the WGCNA method on proteomic data was followed also by MacDonald et al. [18] to clarify the role of the glutamate signaling in schizophrenia (SCZ). The topological evaluation of the co-expression networks from SCZ affected subjects and healthy controls led to observe in SCZ affected group a lower average node degree. This result was probably due to the loss of coordination of the biological functions, as well as disease heterogeneity. However, in SCZ network, it was found the exclusive presence of a module enriched in GO terms related to glutamate signaling and whose proteins had a significant increased degree.

The application of the WGCNA on protein expression profiles was also faced by Chang Guo et al. to characterize the role of different protein isoforms in *E. Coli* resistance to serum killing [13]. Like in other cases, the authors evaluated the topological variations of the co-expression networks between control- and serum-treated groups. By considering the connectivity of modules identified in both networks, a protein, IleS, was found with a differential number of connections in control and treated groups. Of note, its involvement in the response to serum killing was confirmed by independent functional test based on a gene-deletion mutant, thus, confirming the utility to use protein co-expression networks also to identify putative drug targets.

To find phenotype-related biomarkers in the context of renal dysfunction, D. Wu et al. followed an approach based on the combination of differentially expressed proteins and PPI networks. For each pair of connected nodes they calculated the PC score, and the topological analysis of the reconstructed co-expression networks led to identify twelve proteins involved in the pathology [44]. Likewise, Yu et al. investigated the molecular mechanisms underlying the glioblastoma multiforme (GBM)[20]. They analyzed samples of macaque rhesus brain by both iTRAQ and RNA-seq approaches. The proteins identified were combined with STRING database and, for each experimentally validated PPI, the PC score was calculated using both protein and transcript levels. Since the PC score from proteomic data resulted significantly higher than score calculated using transcript levels, the authors focused on WGCNA to identify protein modules involved in the disease. Finally, a more detailed evaluation of these modules allowed the selection of eight genes of interest, and two of them were already known drug targets of GBM.

## Conclusions

The aim of this review was to provide an overview on PPI and co-expression networks. In particular, presenting the recent idea of the protein co-expression networks and their use to infer biological knowledge by topological and module analysis. Although literature is yet too weak, protein co-expression networks represent a valid approach to obtain a novel overview of proteomic data and to provide new hypotheses about key molecules acting in pathophysiological states. Of course, its real value has to be assessed by further studies, but preliminary findings make it promising. The main limitation to perform the construction of protein co-expression networks may be attributed to the difficulty in measuring a proteome with enough coverage. A major consequence is the high rate of missing values that introduce loss of information and significant bias. In addition, batch effects may occur in datasets run in different days or by different technicians. Thus, data normalization is another key point in the context of proteomic data preprocessing. These aspects will be surely improved by future advances of the proteomic technologies which in recent years have received a big boost from genome sequencing and from the combination of liquid chromatography and mass spectrometry [117]. In any case, the availability of large-scale proteomic data already offers a new range of opportunities to improve the existing network models, and in particular PPI, in understanding the mechanisms behind the emergent phenotypes [8, 10, 108, 118, 119].

The results shown through the reviewed studies have evidenced a good relation between the topology of protein co-expression network and the emergent phenotypes. Like PPI networks, the characterization of hubs/bottlenecks and functional, topological and disease modules has proved to select the most important molecules. Despite these findings, statistical methods to construct co-expression networks by processing large-scale proteomic data still need to be improved [63, 64, 66]. To date, the available applications are mainly based on WGCNA framework, and studies evaluating other approaches are expected. Gaussian graphical models [120], partial correlation [121], or Bayesian networks [122] are more sophisticated approaches that are gaining favor over simple correlations due to their ability to separate direct from indirect variable associations. These methods need to use prior knowledge to estimate probabilistic interactions, and their implementation on typical -omics data may be computationally challenging due to the curse of dimensionality. However, they are widely adopted to integrate different -omics data [123, 124] and to infer transcriptional regulatory networks in the context of reverse-engineering processing techniques [48, 49].

Collection and integration of different -omics data represent essential points to perform a global evaluation of the biological systems and to improve the effectiveness of the current systems biology approaches. For these purposes, genomic and proteomic data are often used in combination with PPI networks. Since many studies are reporting a low direct correlation between mRNA and protein abundance [125, 126], their integration with molecules acting in the post-transcriptional regulation [127, 128] and metabolomic data [10] is necessary. In this scenario, PPIs and co-expression networks provide the possibility to apply a multi-omic strategy [15] that should improve level of significance in understanding biological mechanisms, including those related to diseases. Moreover, gene and protein co-expression networks give the opportunity to represent and to evaluate at system level including organisms that lack information about PPIs. In fact, except for human and other few organisms, PPIs are often inferred by homology making incomplete the theoretical models to describe the real-world networks, and with a connectivity affected by false positive interactions [129].

It is evident that the reconstruction of more complete and specific models is key to improve the current systems biology approaches. Molecules and interactions so far considered intracellularly should also be evaluated in tissues, and a new network of relationships that keeps in communication cells, tissues and organs will have to be considered too. On the other hand, computational tools are required to effectively integrate multi-omic experiments [130]. In addition to basic research, these improvements may have important effects into clinical applications opening the way toward the use of multiple biomarkers and their relationships [22–24]. They represent a chance to generate new mathematical models and algorithms for advanced diagnosis and prognosis methods which may lead toward a more preventive, predictive, and personalized medicine [27, 51]. These objectives are the major challenges to be addressed in the near future, and their achievement rely on the synergistic cooperation of biologists, physicists, mathematicians, and bioinformaticians.

## Abbreviations

GBM: Glioblastoma multiforme
GEO: Geometric random graph
GO: Gene ontology
iTRAQ: Isobaric tag for relative and absolute quantitation
LC: Liquid chromatography
MS: Mass spectrometry
PC: Pearson’s correlation
PPI: Protein-protein interaction network
ProCoNa: Protein co-expression network approach
SC: Spearman’s correlation
SCZ: Schizophrenia
SRM: Selected reaction monitoring
TAP-MS: Tandem affinity purification coupled with mass-spectrometry
WGCNA: Weighted gene cp-expression network analysis
Y2H: Yeast two-hybrid
